# Pediatric Hemolytic Uremic Syndrome in North-Eastern Germany During the STEC O45:H2 Outbreak in 2025: Clinical Features and Short-Term Outcomes

**DOI:** 10.1093/ofid/ofag433

**Published:** 2026-07-17

**Authors:** Lea M Merz, Katja Doerry, Florian Buerger, Verena Klämbt, Moritz Boeckelmann, Katalin Dittrich, Emilia Marczak, Michael Pohl, Maria Heyde, Keike Paulsen, Jens Drube, Heimke von Osten, Hagen Staude, Alexander Mellmann, Johannes Holle, Christina Lang, Angelika Fruth, Tanja Jung-Sendzik, Julia Thumfart, Jun Oh, Raphael Schild, Dominik Müller

**Affiliations:** Department of Pediatric Nephrology, University Hospital Leipzig, Leipzig, Germany; Pediatric Nephrology, University Medical Center Hamburg-Eppendorf, Hamburg, Germany; Pediatric Nephrology, University Medical Center Hamburg-Eppendorf, Hamburg, Germany; Department of Pediatric Gastroenterology, Nephrology and Metabolic Diseases, Charité Universitätsmedizin Berlin—Campus Virchow Klinikum, Berlin, Germany; Department of Pediatric Gastroenterology, Nephrology and Metabolic Diseases, Charité Universitätsmedizin Berlin—Campus Virchow Klinikum, Berlin, Germany; Department of Pediatric Nephrology, University Hospital Leipzig, Leipzig, Germany; Department of Pediatric Nephrology, University Hospital Leipzig, Leipzig, Germany; KfH Kidney Center for Children and Adolescents, St Georg Hospital, Leipzig, Germany; Department of Pediatric Nephrology, University Hospital Carl Gustav Carus, Dresden, Germany; Pediatric Nephrology, University Medical Center Hamburg-Eppendorf, Hamburg, Germany; Department of Pediatric Kidney, Liver, Metabolic and Neurological Diseases, Hannover Medical School, Hannover, Germany; Department of Pediatrics, University Hospital Greifswald, Greifswald, Germany; Department of Pediatric Nephrology, University Children's Hospital Rostock, Rostock, Germany; Institute of Hygiene and National Consulting Laboratory for Hemolytic Uremic Syndrome (HUS), University Hospital Münster, Münster, Germany; University Children's Hospital, University Hospital Tübingen, Tübingen, Germany; Department of Infectious Diseases, National Reference Center (NRC) for Salmonella and Other Enteric Pathogens, Robert Koch Institute, Branch Wernigerode, Germany; Department of Infectious Diseases, National Reference Center (NRC) for Salmonella and Other Enteric Pathogens, Robert Koch Institute, Branch Wernigerode, Germany; Department of Infectious Disease Epidemiology, Robert Koch Institute, Berlin, Germany; Department of Pediatric Gastroenterology, Nephrology and Metabolic Diseases, Charité Universitätsmedizin Berlin—Campus Virchow Klinikum, Berlin, Germany; Pediatric Nephrology, University Medical Center Hamburg-Eppendorf, Hamburg, Germany; Pediatric Nephrology, University Medical Center Hamburg-Eppendorf, Hamburg, Germany; Department of Pediatric Gastroenterology, Nephrology and Metabolic Diseases, Charité Universitätsmedizin Berlin—Campus Virchow Klinikum, Berlin, Germany

**Keywords:** children, hemolytic uremic syndrome, serotype O45:H2, Shiga toxin–producing *E. coli*, STEC

## Abstract

**Background:**

Shiga toxin–producing *Escherichia coli* (STEC)–associated hemolytic uremic syndrome (HUS) is a leading cause of pediatric acute kidney injury. Between August and October 2025, Germany experienced an outbreak of the rare STEC serotype O45:H2, comprising 53 laboratory-confirmed pediatric HUS cases. Because O45:H2-associated HUS had previously been reported only sporadically, this study characterized its clinical course and compared it with HUS caused by non-O45:H2 STEC serotypes.

**Methods:**

We retrospectively included all pediatric STEC-HUS cases treated in Northeastern Germany during the outbreak period. Microbiological confirmation was performed through cultivation and molecular typing at the National Reference and Consulting Laboratories. Patients were classified as O45:H2 or non-O45:H2 based on serotyping and the Robert Koch Institute (RKI) O45:H2 outbreak case definition. Clinical and laboratory parameters, dialysis requirements, transfusion needs, and short-term outcomes were compared.

**Results:**

Thirty-seven children with STEC-associated HUS were included: 18 with O45:H2 and 19 with other serotypes. Patients with O45:H2 were significantly younger (median 2.0 vs 4.0 years, *P* = .01), while renal impairment at onset was comparable (minimal estimated glomerular filtration rate 8.0 vs 12 mL/min/1.73 m^2^). Dialysis was required in 65% of patients in both groups, with a trend toward longer duration in O45:H2 cases (median 10 vs 6 days). Hematologic parameters, markers of hemolysis, transfusion needs, neurological symptoms, and intensive care treatment were similar. One child died from myocardial failure. At 3-month follow-up, kidney function had recovered well in both groups.

**Conclusions:**

O45:H2-associated HUS was comparable in severity to non-O45:H2 STEC-HUS, while the younger age of affected children may indicate distinct host susceptibility or exposure patterns.

Hemolytic uremic syndrome (HUS) is a thrombotic microangiopathy defined by microangiopathic hemolytic anemia, thrombocytopenia, and acute kidney injury [1, 2]. It occurs predominantly in young children and is the most common cause of acute intrarenal kidney injury in childhood. In most cases, HUS is triggered by infection with enterohemorrhagic *Escherichia coli*, a highly pathogenic subgroup of Shiga toxin–producing *E. coli* (STEC), with O157:H7 (sequence type (ST) 11 and related STs) being the most extensively studied serotype [1, 2]. STEC infections usually occur sporadically, but larger outbreaks can cause substantial morbidity and mortality also in adolescents and adults, as highlighted by the 2011 European outbreak [3].

In August 2025, an outbreak caused by the STEC serotype O45:H2 originated in the northeastern German state of Mecklenburg-Western Pomerania and subsequently spread to North Rhine-Westphalia, with additional sporadic cases reported in other regions [4, 5]. This outbreak, the largest of its kind in Germany since 2011, currently has an unidentified source, and epidemiological investigations are ongoing [4, 6]. As of 17 November 2025, 199 laboratory-confirmed cases had been attributed to the outbreak, including 53 HUS cases and 145 STEC cases without HUS; disease classification was unknown in 1 case [7]. The median age was 4 years, and all HUS cases occurred in children [7]. Before this outbreak, the STEC causing serotype O45:H2 had been rarely detected in Germany. Between January 2015 and June 2025, the National Reference Centre for Salmonella and Other Bacterial Enteric Pathogens identified only 13 O45:H2 isolates, 4 of them associated with HUS; none were genetically closely related to the O45:H2 outbreak strain [4, 5, 7]. Given the rarity of this serotype and the unusually high number of associated HUS cases, this outbreak provides a unique opportunity to clinically characterize STEC O45:H2-associated HUS and to compare it with non-O45:H2 STEC-HUS cases from the same region and time period.

## METHODS

### Clinical Case Definition

Hemolytic uremic syndrome was defined by the concurrent presence of microangiopathic hemolytic anemia, reflected by hemoglobin concentrations below 10 g/dL (or below local standard values for age) with evidence of erythrocyte fragmentation, thrombocytopenia with platelet counts under 150 × 10^9^/L (or local standard values for age), and acute kidney injury, defined according to the KDIGO pediatric criteria (≥stage 1) [8], with assessment based on age-adjusted reference values in the absence of baseline creatinine measurements. Estimated glomerular filtration rate (eGFR) was calculated using the bedside Schwartz formula (2009), with age- and height-adjusted parameters applied via the PED(z) calculator [9, 10]. Arterial hypertension was defined as blood pressure exceeding the 95th percentile for age and height [11, 12]. Mild neurological symptoms included irritability, lethargy, headache, or mild alterations in mental status, whereas severe neurological involvement comprised seizures, markedly reduced level of consciousness (eg, somnolence or coma), focal neurological deficits, or neurological deterioration requiring intensive care. Fluid management was categorized as infusion, restriction, or monitoring. Infusion was defined as intravenous fluids administered according to weight-based maintenance requirements, calculated using the Holliday–Segar method, with the aim of volume expansion (∼3%–5% weight gain) [13, 14]. Baseline weight was obtained from parental report or health records; if unavailable, volume status was assessed clinically. Restriction was defined as fluid intake below calculated maintenance requirements. Monitoring was defined as observation without active intervention, with oral intake determined by the family.

### Microbiological Confirmation of O45:H2 Cases and Case Definition

The O45:H2 outbreak strain (*stx2a*, *eae* positive) was identified by stool culture followed by confirmatory serotyping. Shiga toxin 2, the *stx2a* and *eae* genes, was detected using immunoassay and/or polymerase chain reaction (PCR) after enrichment of stool samples [15]. All confirmatory diagnostics of the outbreak strain were performed at the Consulting Laboratory for HUS in Münster and the National Reference Centre for Salmonella and Other Bacterial Enteric Pathogens at the Robert Koch Institute (RKI). The RKI outbreak case definition distinguished between confirmed and probable O45:H2 outbreak cases. Confirmed cases included STEC or HUS cases with disease onset on or after 10 August 2025, as well as asymptomatic cases, if laboratory evidence of infection with the outbreak strain was available. Laboratory confirmation was defined as the detection of the O45 antigen by serotyping or O45-wzx–specific PCR together with the detection of *stx2a* and *eaeA* by PCR. Probable cases were defined as cases with disease onset on or after 10 August 2025 and residence in, travel to, or stay in Mecklenburg-Western Pomerania within the 7 days before onset of diarrhea. This category included cases for whom microbiological confirmation was incomplete or no bacterial isolate was available for confirmatory characterization at the Consulting Laboratory for HUS or the National Reference Centre. Based on this definition and the available laboratory results, patients were assigned to either the O45:H2 outbreak group or the non-O45:H2 comparison group. Patients were assigned to the non-O45:H2 comparison group if another STEC serotype was identified, if isolated *stx1* was detected without evidence of *stx2a* or O45 confirmation, or if no serotype was identified but the RKI outbreak criteria were not fulfilled [15–17].

### Data Collection

Data were collected from all 7 pediatric nephrology centers in Northeastern Germany. All patients presenting with STEC-associated HUS between August and October 2025 were included and retrospectively classified as O45:H2 outbreak or non-O45:H2 cases based on laboratory findings; 2 non-O45:H2 cases with symptom onset in June and July were included because of their temporal proximity to the outbreak. In total, clinical data from 37 patients were obtained: 18 were assigned to the O45:H2 group and 19 to the non-O45:H2 group.

### Data Analysis

Patient characteristics were collected, including blood and urine parameters. Median values were calculated, and the interquartile range, defined as the 25th to 75th percentile, was reported to describe the variability of non-normally distributed data. For categorical variables (eg, number of red blood cell and platelet transfusions), proportions were calculated and expressed as percentages. Statistical analyses were performed using GraphPad Prism 10 (Insight Partners, GraphPad Prism, LCC). Data distribution was assessed for both groups using the Kolmogorov–Smirnov test. As most variables were not normally distributed, group comparisons were conducted using the Mann–Whitney *U* test. A 2-sided *P*-value ≤.05 was considered statistically significant. Where appropriate, descriptive statistics were applied.

## RESULTS

**Figure 1.**
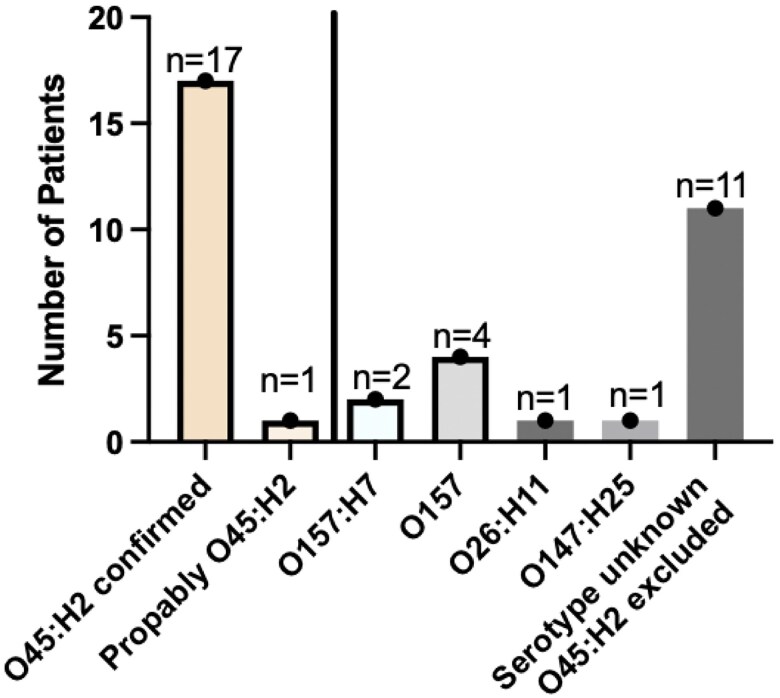
Serotype composition and microbiological classification of included STEC-HUS cases.

Across pediatric nephrology departments in Northeastern Germany, 37 patients with confirmed STEC-associated HUS were identified between August and the end of October 2025. Patients were treated across the participating centers, with most cases managed in Berlin, Hamburg, and Rostock. The O45:H2 outbreak group comprised 17 confirmed outbreak cases and 1 probable case meeting the epidemiological criteria. In the non-O45:H2 comparison group, O157 was the most frequently identified serotype, followed by single cases of O26 and O147. All identified O157, O26, and O147 comparator isolates were *stx2* positive and *stx1* negative. In 11 patients, complete serotype assignment was not possible, or classification was based on exclusion from the outbreak according to the RKI case definition (Figure 1). Of these, 2 were identified as non-O45, 1 was classified as EPEC, and 8 could not be serotyped because no bacterial isolate was available. These 8 patients were assigned to the non-O45 group because they did not meet the epidemiological outbreak criteria, with no reported residence in, travel to, or stay in the affected areas. Overall, Shiga toxin profiles varied, with 8 of 19 cases being *stx2* positive and *stx1* negative, 9 showing combined *stx1*/*stx2* detection or incomplete toxin profiles, and 2 being *stx1* positive only.

### **Clinical** C**haracteristics**

We retrospectively analyzed clinical characteristics of patients in the O45 outbreak group and the non-O45 comparison group (Table 1). Group size and sex distribution were similar. Patients in the O45 group were significantly younger, with a median age of 2.0 years compared with 4.0 years in the non-O45 group (*P* = .01). Markers of acute kidney impairment were broadly comparable between groups, including minimal eGFR, eGFR at discharge, dialysis requirement, and dialysis modality. Dialysis was required in approximately two-thirds of patients in both groups, and peritoneal dialysis was the most frequently used modality. Dialysis duration tended to be longer in the O45 group, although this difference did not reach statistical significance (Table 1).

**Table 1.** Comparison of Clinical Characteristics Between Children With O45:H2-Associated HUS and Those With Other STEC Serotypes

Variable	O45:H2	Non-O45:H2	*P V*alue ((Mann–Whitney *U* or Fisher's Exact Test)
Number of patients	n = 18	n = 19	NA
Sex	M: 14/18 (77.8%) F: 4/18 (22.2%)	M: 14/19 (73.7%) F: 5/19 (26.3%)	NA
Age (in y), median	2.0 (IQR 1.0–3.25)	4.0 (IQR 2–7.0)	*P* = .01
eGFR minimal (mL/min/1.73 m^2^), median	8.0 (IQR 6.0–40.75)	12 (IQR 7.0–72.0)	*P* = .15
eGFR at discharge (mL/min/1.73 m²), median	55.5 (IQR 34.35–91.5)	71.0 (IQR 42.0–100.0)	*P* = .42
Dialysis, n (%)	12/18 (66.7%)	12/19 (63.2%)	*P* > .99
Dialysis modality	PD: 8/12 = 66.7% HD: 4/12 = 33.3%	PD: 7/12 = 58.3% HD: 4/12 = 33.3% HD/PD: 1/12 = 8.3%	NA
Time on dialysis (d), median	10.0 (IQR 7.0–13.5)	6.0 (IQR 3.0–9.75)	*P* = .09
Minimum platelet count (/nL), median	43.0 (IQR 19.25–80.25)	58.0 (IQR 22.0–80.0)	*P* = .63
Minimum hemoglobin (g/dL), median	6.1 (IQR 5.37–6.57)	6.1 (IQR 5.0–6.8)	*P* = .93
Platelet transfusion, n (%)	3/18 (16.7%)	5/19 (26.3%)	*P* = .69
Platelet transfusions	1	1	*P* = .69
Red blood cell transfusion, n (%)	13/18 (72.2%)	14/19 (73.7%)	*P* > .99
RBC transfusions, median	1.5 (IQR 0.75–2.25)	1 (IQR 0–2)	*P* = .71
LDH max (U/L), median	2961 (IQR 1346–3422)	2004 (IQR 1520–2670)	*P* = .33
Hypertension, n (%)	11/18 (61.1%)	8/19 (42.1%)	*P* = .33
Neurological symptoms, n (%)	4/18 (22.2%) only mild symptoms	4/19 (21.1%) total (2 mild, 2 severe)	*P* > .99
ICU admissions, median	11/18 = 61.1%, 4.5 d (IQR 2.25–16.0)	10/19 = 52.6%, 5 d (IQR 3.7–10)	*P* = .74 *P* = .76
Length of hospital duration in days, median	15.5 d (IQR 11.0–19.5)	11 d (IQR 5.0–16.0)	*P* = .09

Continuous variables are given as median (IQR), categorical variables as n (%). Mann–Whitney *U* test was used for group comparisons. NA indicates that no statistical test was applied.

Abbreviations: eGFR, estimated glomerular filtration rate; PD, peritoneal dialysis; HD, hemodialysis; RBC, red blood cell; LDH, lactate dehydrogenase; ICU, intensive care unit; NA, not applicable; IQR, interquartile range.

Hematological involvement was similar in both groups, with red blood cell transfusions required in the majority of patients (72.2% vs 73.7%) and platelet transfusions in a smaller subset (16.7% vs 26.3%). Minimum platelet counts, minimum hemoglobin levels, transfusion requirements, and peak lactate dehydrogenase values did not differ significantly between groups (Table 1). Arterial hypertension occurred in both groups and was observed in 61.1% of O45 patients and 42.1% of non-O45 patients. Neurological symptoms were reported in around one-fifth of patients in each group, with severe neurological involvement observed only in the non-O45 group. More than half of patients required intensive care treatment, and intensive care unit (ICU) stay as well as total hospital stay did not differ significantly between groups (Table 1).

One male patient infected with STEC O157 died during the acute phase of disease. He developed severe thrombotic microangiopathy with multiorgan involvement, including renal, hepatic, cardiac, and neurological impairment. Cerebral imaging showed bilateral basal ganglia diffusion abnormalities, and progressive left ventricular dysfunction culminated in asystole. Despite cardiopulmonary resuscitation and initiation of veno-arterial extracorporeal membrane oxygenation, the patient died from persistent myocardial pump failure. The clinical course was most consistent with severe STEC-associated thrombotic microangiopathy complicated by catastrophic antiphospholipid syndrome, likely triggered by concomitant viral infections.

### **Fluid** M**anagement**

Fluid management varied between centers and patients (Table 2). Overall, 48.6% of children received fluid infusion alone during the acute HUS course, while 21.6% received a combination of infusion followed by restriction. Fluid restriction alone was used in 13.5% of patients, and 16.2% were managed with monitoring only. To further assess the clinical course according to fluid management, we compared dialysis requirements between patients who received infusion therapy and those who did not. Dialysis was required in 16 of 26 patients who received infusion therapy compared with 8 of 11 patients without infusion therapy (61.5% vs 72.7%). This numerical difference did not reach statistical significance (*P* = .7106, Fisher's exact test).

**Table 2:** Overview of Fluid Management Strategies in Children With O45:H2-associated HUS Compared With Other STEC Serotypes

Category	O45:H2 (n = 18)	Non-O45:H2(n = 19)	Total Cohort (n = 37)
Infusion	10 (55.6%)	8 (42.11%)	18 (48.6%)
Both (first infusion and then restriction)	5 (27.8%)	3 (15.79%)	8 (21.6%)
Restriction	2 (11.1%)	3 (15.79%)	5 (13.5%)
Fluid monitoring, no intervention	1 (5.6%)	5 (26.32%)	6 (16.2%)

Values represent n (%) within each group.

### Medication

Only one patient received a single dose of eculizumab because of severe neurological involvement, including status epilepticus, encephalopathy, and magnetic resonance imaging abnormalities. Rasburicase was not administered. Three patients received antibiotics, but none specifically for STEC infection; indications included peritoneal dialysis catheter placement, an initial blood culture positive for *Streptococcus* species, and empiric treatment for elevated inflammatory markers.

### **Three-month** F**ollow-**up

Three-month follow-up at a pediatric nephrology center was available for 77.8% of children in the O45 group and 89.5% in the non-O45 group (Table 3). Hemoglobin levels had normalized and kidney function had recovered well in both groups, with comparable eGFR values at follow-up. Albuminuria patterns were broadly similar, with most children showing no or only mild albuminuria and no cases of severe albuminuria in either group. Persistent arterial hypertension was observed in 28.6% of O45 patients and 5.9% of non-O45 patients, although this difference was not statistically significant (Table 3). A subset of patients, particularly in the O45:H2 group (22.2%), did not receive follow-up at a pediatric nephrology center.

**Table 3:** Comparison of Laboratory and Clinical Findings at 3-Month Follow-up After STEC-HUS in Children with O45:H2 Versus Other Serotypes

Parameter	O45:H2, n = 14/18	Non-O45:H2, n = 17/19	*P V*alue (Mann–Whitney *U* or Fisher's Exact Test)
Hemoglobin, median	12.1 g/dL (IQR 10.55–12.43)	12.0 g/dL (IQR 10.5–12.4)	*P* = .70
eGFR Schwartz, median	133.0 mL/min/1.73 m^2^ (IQR 92.75–157)	125.0 mL/min/1.73 m^2^ (IQR 116–145)	*P* = .82
Albuminuria, median	31.3 mg/g (IQR 0.11–129.5) A1: 4 (28.6%), A2: 5 (35.7%), negative: 5 (35.7%)	12.9 mg/g (IQR 3.55–42.05) A1: 4 (23.5%), A2: 2 (11.8%), negative: 11 (64.7%)	*P* = .86
Hypertension	4/14 = 28.6%	1/17 = 5.9%	*P* = .34

Values are presented as median (IQR) or n (%). Albuminuria (mg/g) categories follow KDIGO A-staging (A1–A2; no A3 detected).

Abbreviations: HUS, hemolytic uremic syndrome; eGFR, estimated glomerular filtration rate; KDIGO, Kidney Disease: Improving Global Outcomes; IQR, interquartile range.

## DISCUSSION

This study aimed to characterize the clinical presentation and short-term renal outcomes of children with HUS during the first reported STEC O45:H2 outbreak in Germany, which occurred in Northeastern Germany between August and October 2025. Given the rarity of this serotype, O45:H2-associated cases were compared with HUS caused by other, more common STEC serotypes. Although the outbreak later extended further into western Germany, the present analysis focuses on the initial cluster within a clearly defined regional and temporal setting. To contextualize the clinical course of O45:H2-associated HUS, we compared 18 affected pediatric patients with 19 children treated for non-O45:H2-associated HUS in the participating centers.

Our results indicate that the clinical severity of O45:H2-associated HUS was similar to that observed in the comparison group. However, interpretation is limited by the relatively small sample size inherent to an outbreak setting and the heterogeneity of the comparison group, which included both laboratory-confirmed non-O45 serotypes (O157, O26, and O147; n = 8) and cases without confirmed serotype assignment, mainly due to limited isolate availability (n = 11). Given this heterogeneity and the limited number of cases per serotype, serotype-specific comparisons within the non-O45:H2 group, including comparisons with O157:H7, were not performed. Affected patients in this outbreak were significantly younger, which may suggest increased susceptibility to STEC O45:H2 in early childhood. However, as neither the infection source nor the transmission route has been identified, the observed age distribution may also reflect a specific exposure setting or transmission pathway that disproportionately affected younger children, potentially in combination with a lower infectious dose required to cause disease in this age group. Together, these limitations indicate that our findings should be interpreted cautiously and primarily as descriptive observations.

The recently published S3 guideline for thrombotic microangiopathy (TMA)/HUS in childhood and adulthood (Living Guideline) does not define fixed thresholds for transfusions or a preferred dialysis modality but emphasizes individualized decision-making based on clinical context and local expertise [18]. This framework may explain the center-specific differences observed in our cohort, particularly with regard to dialysis modality, dialysis setting, and transfusion practice. While one center exclusively used hemodialysis, others used peritoneal dialysis only. In addition, some hospitals performed dialysis exclusively in the ICU, whereas others provided peritoneal dialysis on regular pediatric wards or used chronic dialysis units for hemodialysis when intensive care was not otherwise required. These differences are clinically relevant because they may directly influence management-related outcomes such as ICU stay, dialysis modality, and cumulative red blood cell exposure. Transfusion practice requires particular consideration. From a mechanistic perspective, both erythrocyte and platelet transfusions could potentially exacerbate TMA [18]. Accordingly, platelet transfusion practices were restrictive across all centers and were mainly limited to preparation for invasive procedures, as no major bleeding events occurred. However, red blood cell exposure may still have differed between centers because hemodialysis circuits in 1 center were primed with erythrocyte concentrates. Thus, center-specific management represents a limitation of the present study and should be considered when interpreting outcomes directly related to clinical practice.

The S3 guideline further recommends early volume expansion through intravenous fluid therapy [19–21] to achieve a 3%–5% weight increase within 24–48 hours, aiming to improve renal perfusion and reduce the risk of requiring renal replacement therapy. In our cohort, 70.3% of all children received fluid administration, suggesting that current recommendations for early volume expansion may not yet be fully implemented in clinical practice. Only one patient received eculizumab due to a severe neurological presentation. While eculizumab had historically been used in cases with severe neurological involvement, the new guideline no longer recommends its use, as no evidence for improved outcomes could be demonstrated [19, 22–30].

In our cohort, no significant short-term differences were observed between children in the O45:H2 and non-O45:H2 groups, suggesting that O45:H2-affected patients may not require serotype-specific follow-up strategies beyond established HUS follow-up recommendations. One-fifth of patients were lost to follow-up at pediatric nephrology centers. Although most children recover normal kidney function after HUS, a relevant subset develops long-term renal sequelae, such as hypertension, proteinuria, or reduced kidney function [31]. Accordingly, the current S3 guideline recommends continued monitoring by pediatric nephrologists [19]. Therefore, this loss to follow-up is concerning, as it remains unclear whether these children were at least monitored for potential renal complications by their primary pediatricians or were entirely lost to follow-up, placing them at risk of undetected sequelae.

The recent O45:H2 outbreak underscores that STEC-associated HUS remains a major challenge in pediatric medicine and that pediatric nephrology centers must be prepared for sudden increases in case numbers. Such outbreaks may involve both common and rare STEC serotypes, for which clinical severity, transmission dynamics, and resource requirements may initially be difficult to predict. Comprehensive STEC surveillance, including non-HUS cases, is therefore essential for early outbreak detection, while reporting of HUS cases by medical centers remains an important public health alert mechanism. Given the limited capacity of individual centers, early notification, transparent exchange of case numbers within established center networks, and coordinated patient allocation are critical to ensure adequate care. Beyond acute management, structured follow-up is equally important, as the loss to follow-up observed even within the short observation period is concerning and highlights the need for standardized postdischarge surveillance.

This study provides, to our knowledge, the first systematic analysis of the recent O45:H2 outbreak in children. Our findings suggest that the O45:H2 serotype can lead to severe disease courses similar to those typically associated with common STEC strains. Our findings highlight the clinical relevance of rare STEC serotypes and underscore the need for continued clinical characterization of emerging or uncommon STEC variants in both pediatric and adult populations.
